# International Medical Congress

**Published:** 1881-03

**Authors:** 


### INTERNATIONAL MEDICAL CONGRESS.

Most of our readers are well aware of the fact, that in September last the Executive Committee of the International Medical Congress, issued a call for a meeting of that body in the early part of August of the present year, and the circular showed that the projected arrangements for it were such as would insure its success in both a professional and social point of view.

Another circular—subsequently received—manifested the perfection of further details in its organization, in proof of which we quote the following paragraph:

"It will be the aim of those charged with the arrangements of the Congress to secure for every topic which may be brought forward, attention in proportion to its importance, and consistently with the time at the disposal of the section. But in order to obtain the greatest advantage to our profession from the coming together of so many distinguished men, and to avoid that desultory mode of proceeding by which the fruit of scientific meetings is sometimes wasted, we have thought it desirable to suggest a list (sent herewith) of some special matters for consideration."

The enclosed "list" (above referred to) is classified in various sections, each representing departments of surgery and medicine, and having under each division a definite set of subjects for discussion. For example in Otology the three following topics have been suggested:

1. On the value of operations in which the Tympanic Membrane is incised.

2. On Morbid Growths within the ear, and their treatment.

3. On Loss of Hearing, where the external and middle ears are healthy.

Other departments will undoubtedly have questions offered, equally important in their practical bearings. Any papers or abstracts for presentation must be forwarded to the Secretary before the latter part of April. The Convention will certainly afford a rare opportunity of meeting in an agreeable as well as profitable manner, the medical men of our times, and those who can avail themselves of it cannot fail of exceptional gratification.

